# Synthesis, biological and computational evaluation of novel cyanomethyl vinyl ether derivatives

**DOI:** 10.3389/fphar.2024.1344042

**Published:** 2024-03-04

**Authors:** Endika Martín-Encinas, María Fuertes, Samuel Delgado-Hernández, Fernando García-Tellado, David Tejedor, Concepción Alonso

**Affiliations:** ^1^ Department of Organic Chemistry I, Faculty of Pharmacy and Lascaray Research Center (Lascaray Research Center), University of the Basque Country/Euskal Herriko Unibertsitatea (UPV/EHU), Vitoria-Gasteiz, Spain; ^2^ Instituto de Productos Naturales y Agrobiología, Consejo Superior de Investigaciones Científicas (Spanish National Research Council) Avda, La Laguna, Spain

**Keywords:** cancer, chemotherapy, cyanomethyl vinyl ethers, cytotoxicity, tubulin inhibitors

## Abstract

This work explores the biological evaluation of novel cyanomethyl vinyl ether derivatives as antiproliferative agents. Tubulin, crucial to microtubule structure and function, is a target for cancer therapies. *In vitro* cytotoxicity assessments revealed significant activity in SKOV3 ovarian carcinoma cells and A549 lung carcinoma cells. Structure-Activity Relationship (SAR) analysis indicated that the *E* isomer and specific substitutions influenced the biological activity. Computational assays predicted favorable ADME properties, highlighting potential as anticancerous agents. Molecular docking studies demonstrated that compound **12E**, with the *E* geometry of the double bond and fused polyaromatic rings such as phenanthrene, has robust interaction with tubulin, suggesting enhanced stability due to diverse amino acid interactions. Comparative spatial distributions with colchicine further indicated potential mechanistic similarities.

## 1 Introduction

Cancer represents one of the greatest challenges facing our society today, with nearly 20 million new cases and 10 million deaths being observed by 2022 (GLOBOCAN, 2022). In fact, the development of new drugs against this group of diseases is being addressed for years (Mattiuzzi et al., 2019). Tubulin is a protein critical to the structure and function of microtubules, which are essential components of the cell’s cytoskeleton. It consists of two subunits, alpha and beta, that assemble to form the building blocks of microtubules. These microtubules play a fundamental role in various cellular processes, including cell division, intracellular transport, and cell shape maintenance (Howard et al., 2009). For all this, tubulin is an important target for cancer therapies, as inhibiting its function can disrupt the division of rapidly dividing cancer cells, leading to their death.

Microtubules (MTs) are crucial cellular polymers composed of tubulin dimers, playing pivotal roles in intracellular trafficking, cell morphology, and mitotic spindle assembly (Goodson et al., 2018). Tubulin, a 50 kDa GTP-binding protein, comprises six family members in eukaryotic cells, with α and β tubulins forming cytoplasmic microtubules and ɣ, *δ*, and ε tubulins localizing to the centrosome. Widely distributed, α-β tubulin heterodimers bind GTP, and β tubulin hydrolyzes GTP during microtubule polymerization. ɣ tubulin is involved in nucleating microtubule growth, while *δ*, ε, and ζ tubulins are specific to cilia, flagella, and basal bodies (Findeisen et al., 2014). In humans, 23 functional tubulin genes contribute to the structural complexity of microtubules, characterized by the assembly of α-β tubulin heterodimers into linear protofilaments. Protofilaments laterally associate to form the pseudo-helical structure of microtubules, with most having 13 protofilaments, though variations exist across species (Chaaban et al., 2017).

**FIGURE 1:**
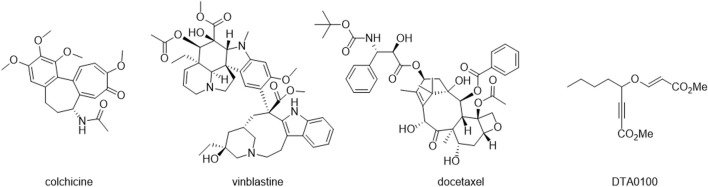
Chemical structures of colchicine, vinblastine, docetaxel and DTA0100.

Microtubule-targeting agents (MTAs) form a diverse group of compounds capable of binding tubulin, influencing microtubule (MT) dynamics by either stabilizing or destabilizing the MT polymer (Steinmetz et al., 2018). Classified into two main categories, MT-stabilizing agents (MSAs) and MT-destabilizing agents (MDAs), these drugs bind to one of seven sites in tubulin dimers. MSAs and MDAs exhibit contrasting effects on MT polymer mass at high concentrations, inducing depolymerization or stabilization. They alter the monomer-to-polymer ratio, impacting cellular functions without significant changes in total MT polymer at low concentrations. Notably, drugs targeting vinca, colchicine, and taxane sites are extensively studied MTAs, historically employed as medicines (Matthew et al., 2021). Colchicine (Figure 1), derived from autumn crocus, is one of the earliest reported MTAs, historically used for gout treatment. Over the past century, MTAs have found applications as herbicides, anti-parasitics, anti-fungal agents, have been explored for neurodegenerative disease and cancer treatment (Graham et al., 1953; Loeffler et al., 1977; Kovacs et al., 2023) and also for antiprotozoal activities (Bethencourt-Estrella et al., 2023; Chao-Pellicer et al., 2023).

After that, various types of tubulin inhibitors, such as vinblastine and docetaxel (Figure 1), have been developed and are used to treat different types of cancer (Kingston, 2001). However, despite tubulin inhibitors have demonstrated significant efficacy in the treatment of cancer, it is important to note that tubulin inhibitors are not specific to cancer cells and can affect healthy cells that divide rapidly, such as bone marrow and gastrointestinal cells. This explains some of the side effects associated with these drugs, such as bone marrow suppression, peripheral neuropathy, and gastrointestinal problems (Dumontet et al., 2010). In this way, computational tools play a pivotal role in discovering new bioactive molecules by enabling precise and rapid calculations of chemical and biological properties. These tools expedite the drug design process by analyzing extensive databases and predicting biological activity, paving the way for the efficient development of compounds with therapeutic potential. (Lipinski et al., 2012; Sliwoski et al., 2014).

Finally, recent investigations have provided compelling evidence regarding the capacity of inhibiting tubulin by compounds with vinyl ethers in their structure. Where, the compound DTA0100 (Figure 1) clearly induced microtubule depolymerization, leading to disturbance of cell cycle kinetics and subsequent apoptosis (Podolski-Renić et al., 2017).

Thereby, in this work we report the biological evaluation of readily available conjugated cyanomethyl vinyl ethers prepared by an organocatalytic multicomponent cyanovinylation of aldehydes recently described by our group (Delgado-Hernández et al., 2021).

## 2 Materials and methods

### 2.1 Chemistry

#### 2.1.1 General experimental information

All reagents from commercial suppliers were used without further purification. All solvents were freshly distilled before use from appropriate drying agents. All other reagents were recrystallized or distilled when necessary. Analytical TLCs were performed with silica gel 60 F254 plates. Visualization was accomplished by UV light. Column chromatography was carried out using silica gel 60 (230–400 mesh ASTM). NMR spectra were obtained on a Bruker Avance 500 MHz spectrometers and recorded at 25°C. Chemical shifts for ^1^H-NMR spectra are reported in ppm downfield from TMS, chemical shifts for ^13^C-NMR spectra are recorded in ppm relative to internal deuterated chloroform (*δ* = 77.2 ppm for ^13^C). Coupling constants (*J*) are reported in Hertz. The terms m, s, d, t, q refers to multiplet, singlet, doublet, triplet, quartet. ^13^C-NMR were broadband decoupled from hydrogen nuclei. High resolution mass spectra (HRMS) was measured by EI method with a Agilent LC-Q-TOF-MS 6520 spectrometer.

#### 2.1.2 Compounds purity analysis

All synthesized compounds were analyzed by HPLC to determine their purity. The analyses were performed on Agilent 1,260 infinity HPLC system (C-18 column, Hypersil, BDS, 5 μm, 0.4 mm × 25 mm). All the tested compounds were dissolved in dichloromethane, and 1 μL of the sample was loaded onto the column. Ethanol and heptane were used as mobile phase, and the flow rate was set at 1.0 mL/min. The maximal absorbance at the range of 190–625 nm was used as the detection wavelength. The purity of all the tested compounds is >95%, which meets the purity requirement by the Journal.

#### 2.1.3 Synthesis of cyanomethyl vinyl ethers

All compounds were synthesized using the methodology described in reference (Delgado-Hernández, 2021) except compound **3** and **4** which are described below.

##### 2.1.3.1 General procedure for the synthesis of cyanomethyl vinyl ethers 3 and 4

To a solution of benzaldehyde (2.0 mmol), acetone cyanohydrin (2.0 mmol) and the corresponding propiolate derivative (2.0 mmol) in *n*-hexanes (6 mL) was added *N*-methylmorpholine (0.05 mmol) at once and the reaction mixture was stirred for 1 h at room temperature. The solvent was removed under reduced pressure, and the residue was purified by flash column chromatography (silica gel; *n*-hexane/ethyl acetate: 80/20) to give the desired 3-(cyanomethoxy)acrylate.

**Propyl (*E*)-3-(cyano(phenyl)methoxy)acrylate** (**3E**). (438.2 mg, 46%). White solid: ^1^H NMR (CDCl_3_, 400 MHz): 0.94 (t, 3H, ^3^ *J*(_HH_) = 7.4 Hz), 1.62–1.71 (m, 2H), 4.08 (t, 2H, 3*J*(_HH_) = 607 Hz), 5.53 (d, 1H, ^3^ *J*(_HH_) = 12.6 Hz), 5.64 (s, 1H), 7.46–7.53 (m, 5H), 7.55 (d, 1H, ^3^ *J*(_HH_) = 12.6 Hz). ^13^C NMR (CDCl_3_, 100 MHz): *δ* = 10.4, 22.0, 66.0, 70.2, 101.6, 115.3, 127.5 (2C), 129.4 (2C), 130.8, 131.2, 157.9, 166.4 ppm. HRMS (ESI-): m/z [M]- calculated for C_14_H_15_NO_3_ 244.0974, found 244.0979.

**Propyl (*Z*)-3-(cyano(phenyl)methoxy)acrylate** (**3Z**). (323.0 mg, 33%). White solid: ^1^H NMR (CDCl_3_, 400 MHz): 0.92 (t, 3H, 3*J*(_HH_) = 7.4 Hz), 1.60–1.69 (m, 2H), 4.06 (t, 2H, ^3^ *J*(_HH_) = 607 Hz), 5.08 (d, 1H, ^3^ *J*(_HH_) = 7.1 Hz), 5.81 (s, 1H), 6.59 (d, 1H, ^3^ *J*(_HH_) = 7.0 Hz), 7.42–7.44 (m, 3H) 7.53–7.56 (m, 2H). ^13^C NMR (CDCl_3_, 100 MHz): *δ* = 10.4, 22.0, 65.7, 72.0, 101.4, 115.6, 127.4 (2C), 129.4 (2C), 130.6, 131.3, 152.6, 164.4 ppm. HRMS (ESI-): m/z [M]- calculated for C_14_H_15_NO_3_ 244.0974, found 244.0979.

**Isoropyl (*E*)-3-(cyano(phenyl)methoxy)acrylate (4E)**. (382.52 mg, 52%). Colorless oil: ^1^H NMR (CDCl_3_, 400 MHz): 1.25 (5, 6H, ^3^ *J*(_HH_) = 6.2 Hz), 5.02–5.09 (m, 1H), 5.50 (d, 1H, ^3^ *J*(_HH_) = 12.6 Hz), 5.63 (s, 1H), 7.46–7.53 (m, 5H), 7.54 (d, 1H, ^3^ *J*(_HH_) = 12.6 Hz). ^13^C NMR (CDCl_3_, 100 MHz): *δ* = 21.9 67.7, 70.1, 102.0, 115.3, 127.4 (2C), 129.4 (2C), 130.7, 131.2, 157.8, 165.9 ppm. HRMS (ESI+): m/z [M + Na]+ calculated for C_14_H_15_NO_3_ 268.0950, found 268.0944.

**Isoropyl (*Z*)-3-(cyano(phenyl)methoxy)acrylate** (**4Z**). (259.8 mg, 35%). Light yellowish oil: ^1^H NMR (CDCl_3_, 400 MHz): 1.23 (5, 6H, ^3^ *J*(_HH_) = 6.2 Hz), 5.00–5.07 (m, 1H), 5.06 (d, 1H, ^3^ *J*(_HH_) = 7.0 Hz), 5.82 (s, 1H), 6.57 (d, 1H, ^3^ *J*(_HH_) = 7.0 Hz), 7.42–7.44 (m, 3H) 7.53–7.56 (m, 2H). ^13^C NMR (CDCl_3_, 100 MHz): *δ* = 21.8, 67.3, 71.9, 101.9, 115.7, 127.3 (2C), 129.3 (2C), 130.5, 131.4, 152.4, 163.6 ppm. HRMS (ESI+): m/z [M + Na]+ calculated for C_14_H_15_NO_3_ 268.0950, found 268.0944.

### 2.2 Biology

#### 2.2.1 Materials

Reagents and solvents were used as purchased without further purification. All stock solutions of the investigated compounds were prepared by dissolving the powered materials in appropriate amounts of DMSO. The final concentration of DMSO never exceeded 5% (v/v) in reactions. Under these conditions DMSO was also used in the controls and was not seen to affect tested compounds activity. The solutions were stored at 5°C until they were used.

#### 2.2.2 Cytotoxicity assays

Cells were cultured according to the supplier´s instructions (ATCC technologies, A-549 (CCL-185), BT-20 (HTB-19), SKOV3 (HTB-77), HCT116 (CCL-247), MRC5 (CCL-171). Cells were seeded in 96-well plates at a density of 2–2.5 × 10^3^ cells per well and incubated overnight in 0.1 mL of media supplied with 10% Fetal Bovine Serum (Lonza) in 5% CO_2_ incubator at 37°C. On day 2, drugs were added and samples were incubated for 48 h. After treatment, 10 µL of cell counting kit-8 was added into each well for additional 2 h incubation at 37°C. The absorbance of each well was determined by an Automatic Elisa Reader System at 450 nm wavelength.

### 2.3 Computational assays

#### 2.3.1 *In silico* ADME

The physicochemical and pharmacokinetics properties of tested compounds were calculated using swissADME (http://www.swissadme.ch/) and pkCSM (http://biosig.unimelb.edu.au/pkcsm/) online web servers. Chemical structures were imported in swissADME and pkCSM tools to calculate molecular as well as ADME properties of the compounds (Daina et al., 2017; Bakchi et al., 2022).

#### 2.3.2 *Docking* studies

Tubuline complex for the docking in the Protein Data Bank (PDB). The X-ray structure code 1SA0 (3.58 Å resolution) was chosen, a tubuline domain complex with colchicine as a ligand. Maestro (Frisch et al., 2016) graphic interface was used, and the Glide 6.9 application (Schrodinger Release, 2015-1: Glide, 2015a) in XP mode (extraprecision) (Friesner et al., 2006) was chosen for the docking. The grid was set up in a box of 20 × 20 × 20 Å, centered in the geometric center of colchicine (β-Chain). The colchicine region in the active site was selected as the target for the screening. The protein complex was optimized and minimized using the Protein Preparation Wizard panel of Schrodinger Suites 2015.1 (Schrodinger Release, 2015-1: Protein Preparation Wizard, 2015b). Likewise, the structures of the different ligands to be interacted with protein and the ligand initially present in the complex, colchicine, were prepared. The binding orders and the protonation states of residue were corrected. The complex previously indicated and used for the different docking processes.

## 3 Results

### 3.1 Chemistry

**SCHEME 1:**
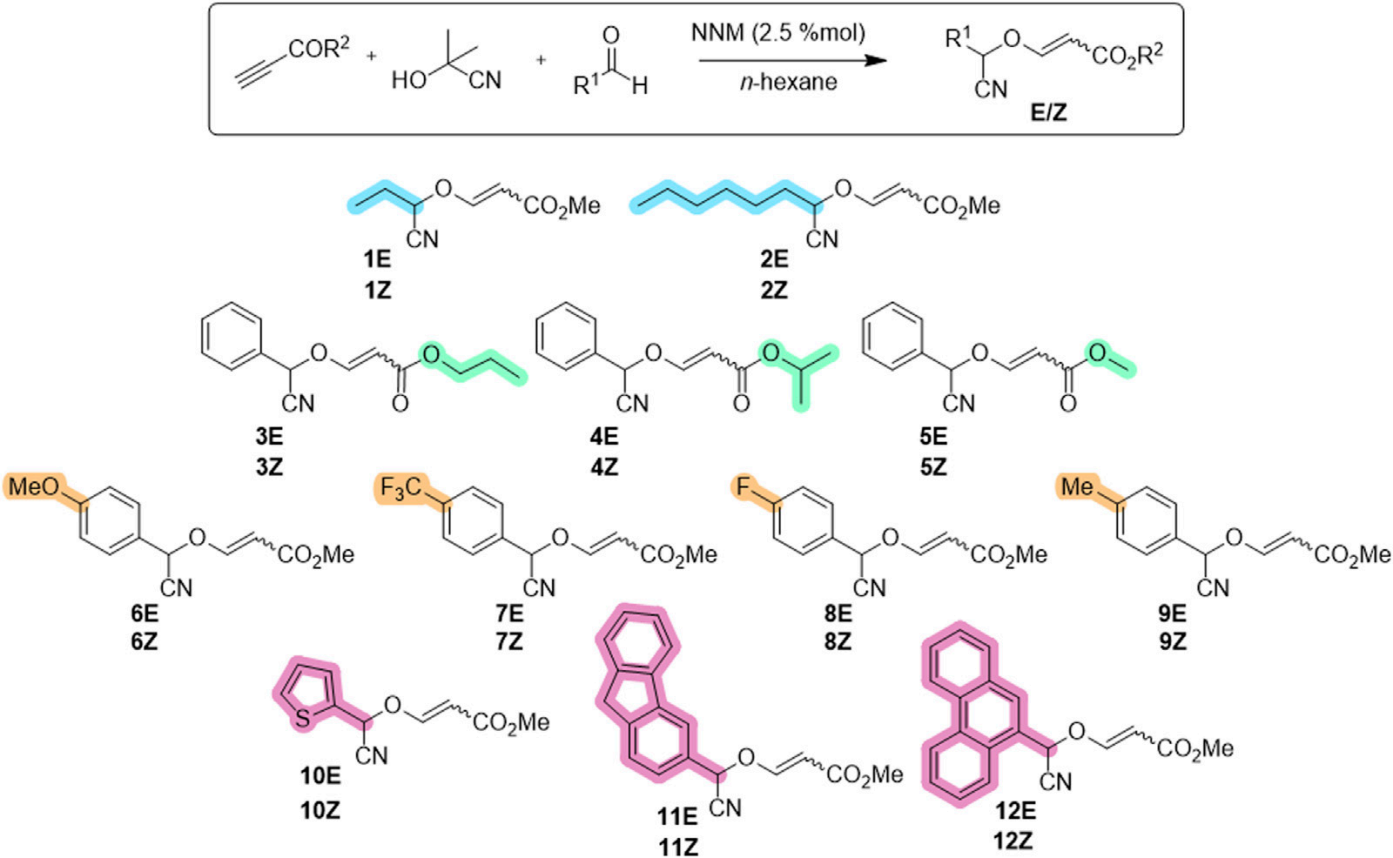
Chemical stluctures of synthesized cyanovinyl ether derivatives **1-12**.

The compounds used for this study were chosen from the set of compounds synthesized in our previous study or were synthesized for the first time to include carboxylic esters with longer alkyl chains (**3**–**4**). Thus, the reaction was implemented for the synthesis of these 3-substituted 3-(cyanomethoxy)acrylates, using aldehydes as substrates, acetone cyanohydrin as the cyanide anion source, and commercially available methyl propiolate or readily synthesized alkyl propiolates as the source of the vinyl component. The multicomponent reaction is catalyzed by *N*-methylmorpholine (2.5 mol%) to deliver the desired 3-(cyanomethoxy)acrylates in excellent yields (84%–95% for isomer mixtures; 32%–56% for separated isomers) for their biological evaluation (Scheme 1).

### 3.2 Biology

#### 3.2.1 *In Vitro* cytotoxicity

The cytotoxicity of the new synthesized 3-(cyanomethoxy)acrylates was investigated *in vitro* by testing the antiproliferative activities against two human cancer cell lines: A549 (carcinomic human alveolar basal epithelial cells), SKOV3 (human ovarian carcinoma), BT20 (human breast carcinoma), HCT-116 (human colorectal carcinoma) and MRC-5 (non-malignant lung fibroblasts). The cell counting kit (CCK8) assay was employed to assess growth inhibition and, the cell proliferation inhibitory activities of the compounds are listed in Table 1 as IC_50_ values (Table 1).

**TABLE 1:** Antiproliferative activities of tested compounds.

Entry	Compound	IC50 (µM)				
		SKOV3	BT20	A549	HCT116	MRC5
1	**COL**	11.58 ± 2.33	21.98 ± 1.30	2.39 ± 1.40	9.32 ± 0.2	5.72 ± 2.1
2	**1E**	2.92 ± 0.28	7.07 ± 1.28	>50	>50	>50
3	**1Z**	>50	>50	>50	>50	>50
4	**2E**	41.58 ± 2.46	15.83 ± 2.90	>50	>50	>50
5	**2Z**	>50	17.28 ± 3.44	>50	>50	>50
6	**3E**	>50	ND	>50	>50	>50
7	**3Z**	>50	ND	>50	>50	>50
8	**4E**	>50	ND	>50	>50	>50
9	**4Z**	>50	ND	>50	>50	>50
10	**5E**	3.84 ± 1.56	8.31 ± 1.26	18.85 ± 1.20	>50	>50
11	**5Z**	29.81 ± 2.09	19.92 ± 3.42	>50	>50	>50
12	**6E**	12.44 ± 1.18	27.79 ± 5.31	>50	>50	>50
13	**6Z**	31.33 ± 4.42	12.61 ± 1.76	>50	>50	>50
14	**7E**	2.62 ± 0.53	10.02 ± 2.17	29.34 ± 1.10	>50	>50
15	**7Z**	>50	>50	>50	>50	>50
16	**8E**	>50	ND	>50	>50	>50
17	**8Z**	>50	ND	>50	>50	>50
18	**9E**	8.08 ± 2.08	4.98 ± 2.77	21.38 ± 0.83	>50	>50
19	**9Z**	>50	20.24 ± 3.78	>50	>50	>50
20	**10E**	>50	ND	>50	>50	>50
21	**10Z**	>50	ND	>50	>50	>50
22	**11E**	28.85 ± 2.15	21.67 ± 0.67	>50	>50	>50
23	**11Z**	38.29 ± 2.31	>50	>50	>50	>50
24	**12E**	7.35 ± 1.42	7.0 ± 1.96	9.89 ± 0.25	16.10 ± 1.20	14.39 ± 0.86
25	**12Z**	11.93 ± 1.88	11.94 ± 1.10	>50	>50	>50

ND, not determined. COL, colchicine. Bold values are numbers of synthesized compounds.

Firstly, selective cytotoxicity was observed in SKOV3 and BT20 cell lines, where most of the compounds showed cytotoxicity. Notably, the best results were observed for compounds **1E** and **7E** with IC_50_ values of 2.92 ± 0.28 μM and 2.62 ± 0.53 μM, respectively in the SKOV3 cell line (Table 1, entries 2 and 14) and for compound **9E** in the BT20 line with IC_50_ of 4.98 ± 2.77 μM (Table 1, entry 18).

**FIGURE 2:**
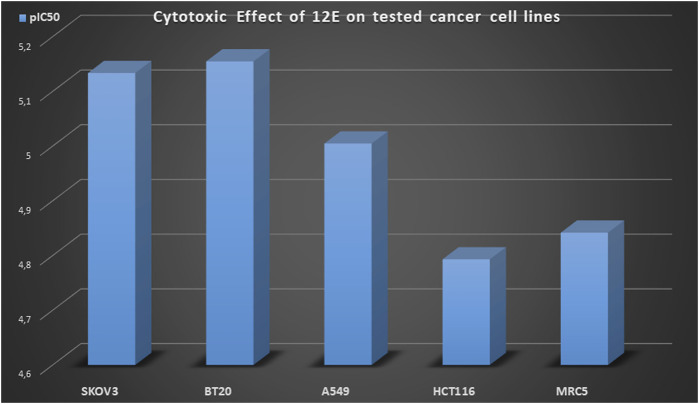
Cytotoxic effect (pIC_50_ values) of cyanovinyl ether derivative **12E**.

On the other hand, only four of the compounds studied showed cytotoxic activity against the A549 line, where compound **12E** obtained the best result with an IC_50_ value of 9.89 ± 0.25 μM (Table 1, entry 24). Precisely this compound **12E** showed interesting cytotoxicity in all the cell lines studied. Besides the above mentioned result on the A549 cell line, it also showed second best result among all the studied compounds on the BT20 cell line with an IC_50_ = 9.89 ± 0.25 and in HCT116 cell line with an IC_50_ = 16.10 ± 1.20 (Figure 2; Table 1, entry 24).

And remarkably, except for compound **12E**, which showed cytotoxicity in all cell lines, none of the tested compounds showed cytotoxicity in the non-cancerous line MRC5. Such behavior against the non-cancerous line MRC5 is relevant, as this selectivity is not found in colchicine, with IC_50_ values = 5.72 ± 2.1 μM in the non-cancerous cell line MRC5 (Table 1, entry 1).

### 3.3 Structure-activity relationship (SAR)

**FIGURE 3:**
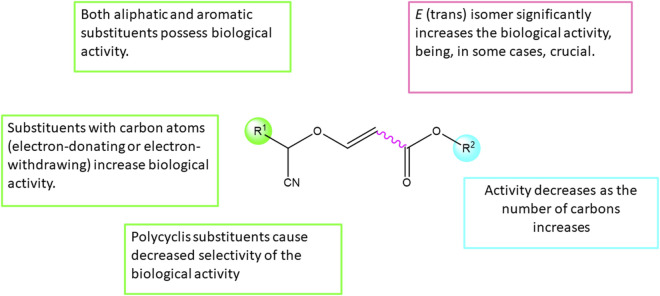
Structure-Activity Relationship (SAR) of synthesized cyanovinyl ethers.

Structure-Activity Relationship (SAR) analysis is a fundamental approach that investigates the links between the structure of chemical compounds and their observed biological or physicochemical properties. By systematically studying the relationships between structural features and activity, SAR analysis provides valuable insights for rational compound design and optimization. The structure-activity relationship of the compounds observed in this study is described (Figure 3).

Based on the obtained biological results and taking into account the structural differences of the tested compounds, it has been observed that the *E* isomer of the tested derivatives significantly increases the biological activity, being even, in some cases, crucial for the compound to exhibit cytotoxicity.

Furthermore, it has been observed that substituents in the ether group (R^2^) can significantly modify the activity of the tested derivatives, where the methyl group (R^2^ = Me) appears to be crucial for the biological activity of the compound (Table 1, entries 6, 8 and 10).

Finally, the nature of the substituents in α position respect to the cyano group (R^1^) has no great effect on the biological activity, observing cytotoxicity with both aromatic and aliphatic groups (Table 1, entries 2 and 10). Furthermore, in the case of aromatic groups, on the one hand, derivatives with a carbon atom at position 4 of the phenyl group show increased cytotoxicity (Table 1, entries 12, 14 and 18), while on the other hand, polyaromatic substituents show loss of selectivity against non-tumorigenic cells (Table 1, entry 24).

### 3.4 Computational assays

#### 3.4.1 In silico ADME

Computer based ADME studies, also known as computer-based studies, play a crucial role in the drug discovery and development process. ADME stands for Absorption, Distribution, Metabolism, and Excretion, which are key factors that determine the pharmacokinetics and efficacy of a drug. In silico ADME studies involve the use of computational models and algorithms to predict the ADME properties of a drug candidate before it is tested *in vitro* or *in vivo*. These studies not only save time and resources but also help in the identification of potential safety concerns associated with a drug candidate.

On the one hand, molecular properties such as partition coefficient (Log Po/w), molecular weight, hydrogen bond donors and acceptors, topological polar surface area (TPSA), violation of Lipinski rule of five were assessed (Table 2).

**TABLE 2:** ADME properties of tested compounds.

Entry	Compound	LogP	Mol. Wt	H-donor	H-acceptor	TPSA	Lipinski #violations
1	**COL**						
2	**1**	2.00	169.18	0	4	59.32	0
3	**2**	3.06	225.28	0	4	59.32	0
4	**3**	2.81	245.27	0	4	59.32	0
5	**4**	2.50	245.27	0	4	59.32	0
6	**5**	2.39	217.22	0	4	59.32	0
7	**6**	2.57	247.25	0	5	68.55	0
8	**7**	2.55	285.22	0	7	59.32	0
9	**8**	2.30	235.21	0	5	59.32	0
10	**9**	2.52	231.25	0	4	59.32	0
11	**10**	2.29	223.25	0	4	87.56	0
12	**11**	2.98	305.33	0	4	59.32	0
13	**12**	3.18	307.34	0	4	59.32	0

COL, colchicine. Bold values are numbers of synthesized compounds.

In general, a drug candidate with a LogP value between 0 and 5 is considered to have favorable ADME properties. A LogP value that is too low may indicate poor lipid solubility, which can affect the absorption and distribution of the drug in the body. On the other hand, a LogP value that is too high may indicate poor aqueous solubility, which can lead to poor bioavailability and potential toxicity due to the accumulation of the drug in fatty tissues. In the case of our compounds, they have a LogP value between 2.0 and 3.18, so they would fall within the favorable range mentioned above (Table 2).

TPSA, or the Topological Polar Surface Area, is another commonly used parameter in drug discovery and development to predict the ADME properties of a drug candidate. TPSA is a measure of the polar surface area of a compound, which is important for its interaction with biological targets and its ability to cross biological membranes. In general, a drug candidate with a TPSA value between 20 and 140 Å^2^ is considered to have favorable ADME properties. As shown in Table 2, all the compounds tested in this work fall within the favorable range for good oral bioavailability.

Finally, the Lipinski’s Rule of Five, a widely used rule in drug discovery to assess the drug-like properties of a compound, was performed to study our compounds. It is recommended that the orally active drug candidate should not have more than one violation of the Lipinski’s rule. As shown in Table 2, all compounds tested meet the established criteria.

On the other hand, the absorption of a drug is a critical factor in determining its efficacy and safety (Supplementary Table S1). It refers to the process by which a drug enters the bloodstream and reaches its target site of action. Understanding the factors that influence drug absorption is essential for optimizing drug therapy and minimizing adverse effects. Because of this, the absorption of drug was evaluated based on aqueous solubility, intestinal absorption and permeability showed in Supplementary Table S1. Thus, the aqueous solubility (Log S) of all compounds ranges from −4.944 to −0.757 log mol/L, which shows the moderate solubility in water of the synthesized compounds. In addition, all of compounds show intestinal absorption above 95% that is interesting due to the most orally administered drugs are primarily absorbed through small intestine due to its large surface area. Finally, the predicted Caco-2 permeability, the logarithm of the apparent permeability coefficient (log Papp >8 × 10^−6^ cm/s), was studied. In our case, all of the synthesized compounds have high Caco-2 permeability taking into account that their predicted value is >0.90.

Lastly, the synthesized compounds volume of distribution (VDss), blood-brain barrier permeability (BBB permeability) and the fraction of unbound was further assessed (Supplementary Table S1). On the one hand, taking into account that the predicted value of Log VDss >0.45 L/kg indicates higher volume of drug distribution, we can say that all synthesized compounds have low volume of distribution in tissues. On the other hand, blood-Brain Barrier (BBB) property is crucial for the effectiveness of drugs in treating central nervous system disorders. The compound is said to be easily permeable through BBB if the predicted value of log BB is > 0.3 and poorly distributed if the value is < -1. It is interesting to note that all compounds show excellent BBB parameters.

Cytochrome P450 (CYP) enzymes are a family of heme-containing enzymes involved in the metabolism of a wide range of endogenous compounds and xenobiotics. These enzymes play a crucial role in drug metabolism, and their activity can influence the efficacy and safety of many therapeutic agents. The compounds were studied as possible CYP2D6, CYP3A4, CYP1A2, CYP2C19, and CYP2C9 enzyme inhibitors (Supplementary Table S1). It is noteworthy that the vast majority of compounds (3, 5, 7, 8, 9, 10, 11 and 12) show the ability to inhibit the CYP1A2 enzyme, and in addition, only compounds 11 and 12 show the ability to inhibit the cytochrome CYP2C9 and CYP2C19 which are primarily expressed in the liver and plays a critical role in the metabolism of numerous drugs, including nonsteroidal anti-inflammatory drugs (NSAIDs), anticoagulants, and antidiabetic agents. Summarizing, the total clearance is primarily a combination of hepatic as well as renal clearance and is measured by the proportionality constant CLtot in log(mL/min/kg). The predicted value of all the synthesized compounds shows lower CLtot ranging from 0.427 to 1.863.

**FIGURE 4:**
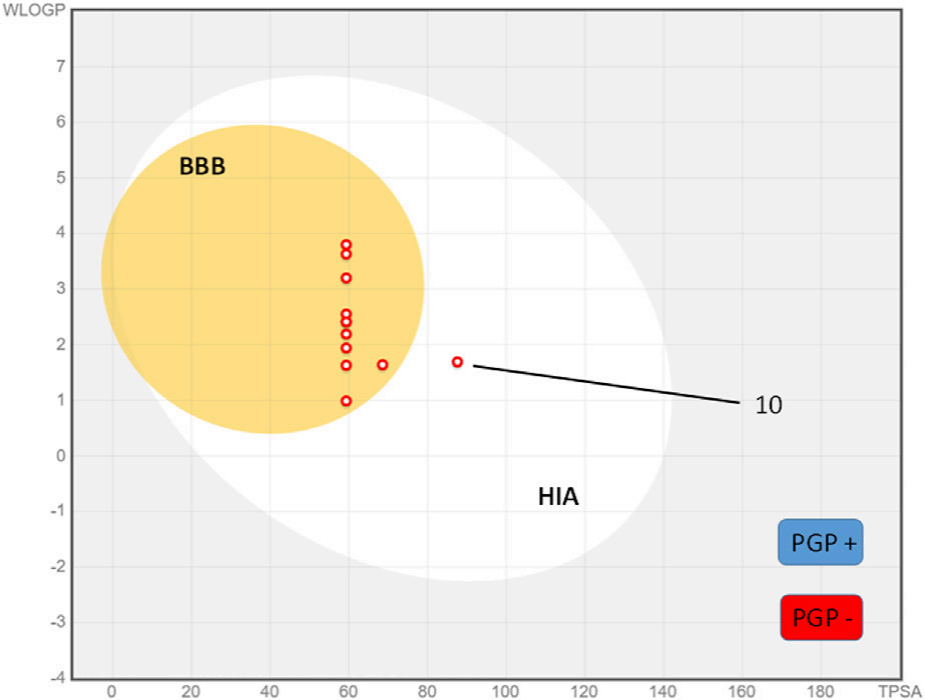
Boiled egg model of synthesized cyanovinyl ethers **1-12**.

The boiled egg model is a simple and inexpensive method that has been used to predict the ADME parameters of drugs. This model involves the use of a boiled egg as a surrogate for the human body, and the measurement of the drug’s permeability through the eggshell and its distribution within the egg. Figure 4 shows the results of the compounds synthesized in a boiled egg projection where the egg yolk corresponds to both the blood-brain barrier (BBB) and human gastrointestinal absorption (HIA) compounds and the egg white only to the HIA-positive compounds. As can be seen in the boiled egg, all compounds tested have positive BBB permeability and HIA, except compound 10 which only show positive HIA.

On the other hand, the representation shows a classification of the compounds by color according to their relationship with the P-glycoprotein (PGP). Point colored in blue are for molecules predicted to be substrate of the P-glycoprotein (PGP+) and hence actively pumped up from the brain or to the gastrointestinal lumen. If predicted non-substrate of the P-glycoprotein (PGP-), the related point is in red. When a compound is identified as a substrate for P-glycoprotein (PGP), it signifies the molecular recognition by PGP of said compound as a species earmarked for active extracellular transport. This discernment carries profound ramifications for the pharmacokinetic profile of pharmaceutical agents. If a drug emerges as a PGP substrate, its processes of absorption, distribution, and elimination stand susceptible to modulation by the dynamic activity of this integral membrane protein (Gottesman et al., 2002). In the case of our tested compounds, it should be noted that all derivatives present the necessary conditions to be PGP-substrate candidates (Figure 4). Concerning our tested compounds, it is worth noting that none meet the necessary criteria to be considered substrates of P-glycoprotein (PGP). As a result, these compounds will not be expelled from the cell, potentially increasing their cytotoxic effects.

#### 3.4.2 Molecular docking

Molecular docking has emerged as a powerful computational tool in drug discovery and structural biology. This technique plays a crucial role in predicting the binding mode and affinity between a protein target and small molecule ligands. By simulating the interactions at the atomic level, molecular docking enables researchers to understand the underlying molecular mechanisms and optimize the design of potential therapeutic compounds.

**FIGURE 5:**
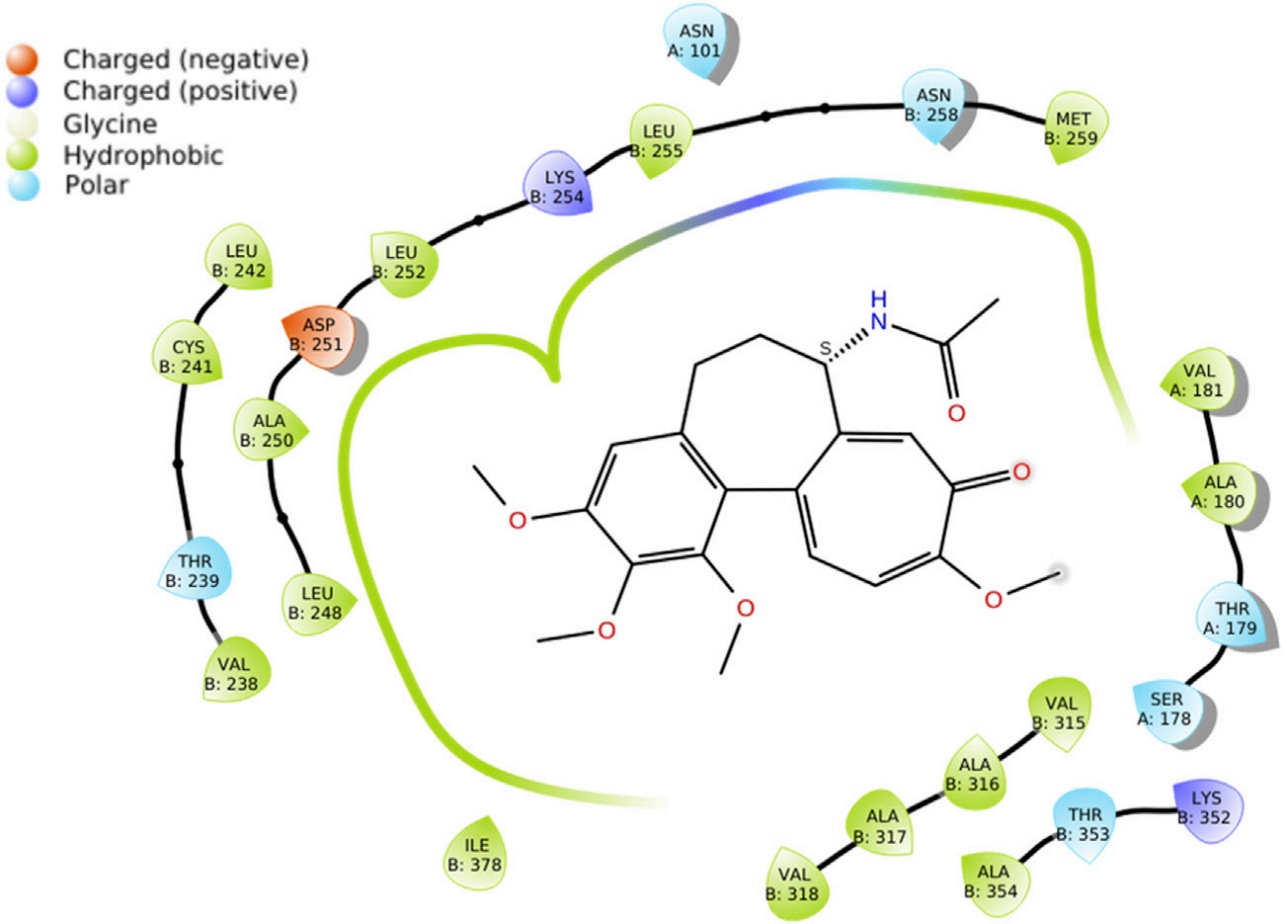
Docking studies of S-colchicine.

The crystallographic data of tubulin in complex with modify colchicine was retrieved from the Protein Data Bank (PDB ID: 1SA0). As it can be observed in Figure 5 docking studies of *S*-colchicine demonstrated robust interactions with crucial amino acid residues, such as Leu248, Asp251, Leu255, and Val260, within the colchicine-binding site of tubulin. Consequently, these amino acid residues played a pivotal role in facilitating inhibitor binding.

Among all the compounds synthesized in this work, a molecular docking study was carried out to investigate its plausible binding pattern and its interaction with the key amino acids in the active site of the protein. The results obtained from molecular docking studies (Table 3) are presented specifically focused on the ‘gScore’ and ‘geMode’ parameters. On the one hand, ‘gScore’ parameter serves as the comprehensive affinity score calculated through docking algorithms, reflecting the overall interaction strength between a target molecule and a ligand with a low ‘gScore’ correlates with an increased likelihood of a stable interaction. On the other hand, the ‘geMode’ pertains to the global energy model, offering an assessment of the quality and stability of the resulting docking configuration. This parameter provides insights into the energetics governing the ligand-receptor interaction.

**TABLE 3:** Docking studies of tested compounds.

Entry	Compound	*E* Isomer		*Z* Isomer	
		gScore (kcal/mol)	geMode (kcal/mol)	gScore (kcal/mol)	geMode (kcal/mol)
1	**1**	−3.685	−27.533	−3.227	−26.279
2	**2**	1.113	−31.899	−3.102	−31.099
3	**3**	−4.064	−41.695	−5.415	−38.099
4	**4**	−4.110	−41.695	−5.484	−43.077
5	**5**	−4.872	−37.238	−4.712	−37.384
6	**6**	−5.922	−40.470	−5.459	−41.437
7	**7**	−5.920	−40.470	−5.866	−41.968
8	**8**	−5.176	−36.886	−4.572	−35.867
9	**9**	−5.564	−39.392	−5.492	−36.957
10	**10**	−4.357	−38.398	−4.039	−32.436
11	**11**	−5.878	−49.124	−7.247	−51.923
12	**12**	−7.733	−66.097	−6.844	−52.137
13	**COL**	−7.578 **gScore (kcal/mol)**		−57.983 **geMode (kcal/mol)**	

COL, colchicine. Bold values are numbers of synthesized compounds.

Thus, a molecular docking study examined the interaction of compounds **1**–**12** with tubulin, taking into account both *E* and *Z* isomeric forms. There were observed variances in the gScore and geMode values across various compounds, as shown in Table 3.

In the first place, it is noteworthy that the average energy of geMode for *E* isomer (−39.931 kcal/mol) is lower than the average energy of compounds with *Z* isomerism (−37.742 kcal/mol). In other words, compounds with *E* isomerism tend to form a more stable protein-inhibitor complex, in general. For example, compound **8**, in its *E* and *Z* forms, exhibited a substantial difference in gScore (−5.176 vs. −4.571 kcal/mol) and geMode (−36.886 vs. −35,867 kcal/mol), highlighting a pronounced isomer-dependent effect on binding affinity.

In this context, it is noteworthy to emphasize that among the range of compounds evaluated, the one that stands out for exhibiting the most intense and sustained interaction with the protein is compound **12E** (Table 3, entry 12). This compound demonstrates a remarkable gScore of −7.733 kcal/mol, indicative of an exceptionally high affinity for the target protein. The pronounced stability of this interaction suggests that the *E* isomer of **12E** is highly conducive to form a robust protein-inhibitor complex, potentially translating into enhanced efficacy as an inhibitory agent. Additionally, it is worth noting that this compound exhibited cytotoxic activity across all tested cell lines.

**FIGURE 6:**
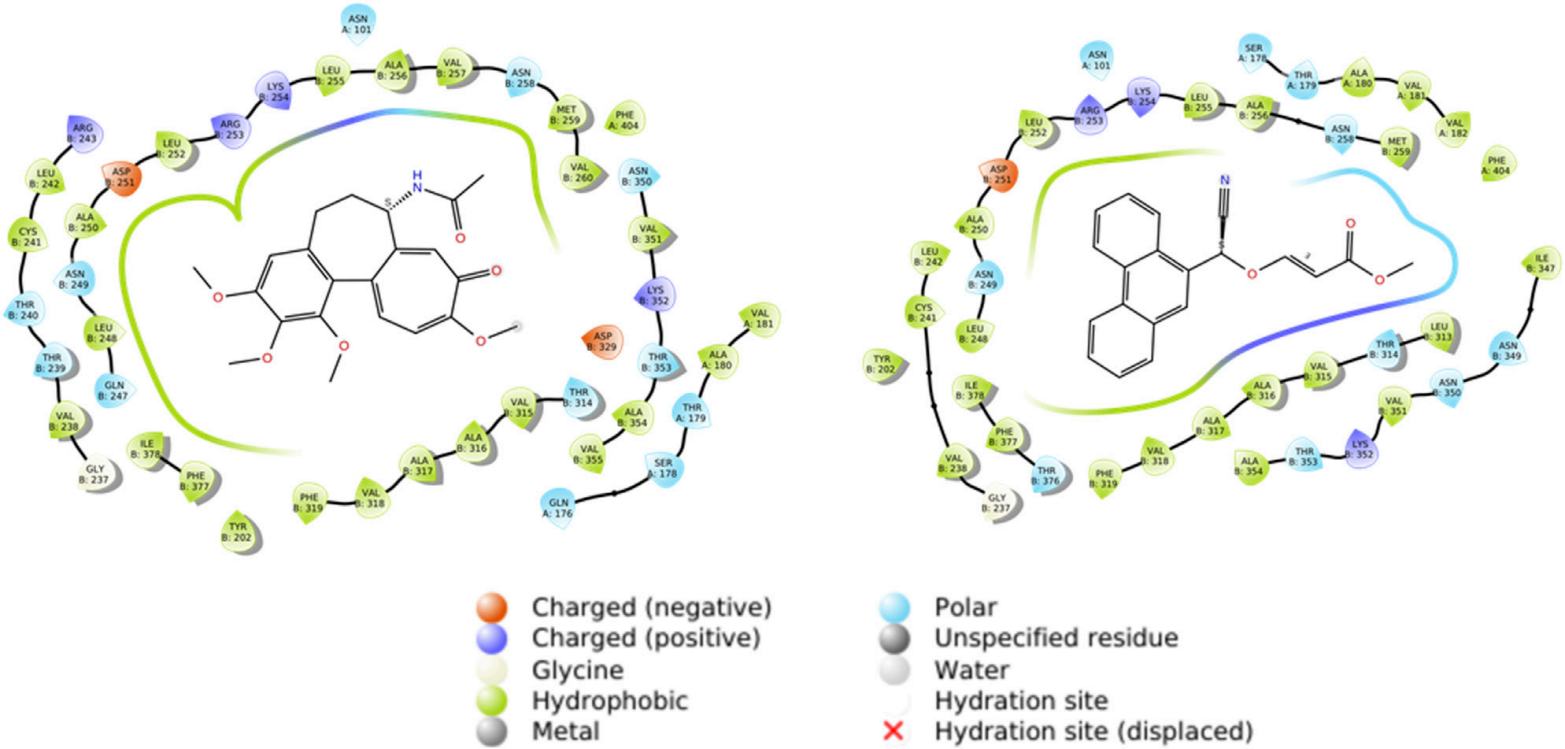
Docking studies of colchicine and compound **12E**.

The stability values exhibited by the tubulin-compound **12E** complex may be attributed to the formation of tight interactions with various amino acids of the protein. As observed in Figure 6, both colchicine and compound **12E** show hydrophobic interactions with residues such as Leu252, Asp251, and Leu255 of tubulin, among others (Table 4). However, in addition to these interactions, compound **12E** presents weak polar interactions with residues like Met259 and Leu313 (Table 4).

**TABLE 4:** Type of interaction in molecular docking.

Residues (Chain B)	Bond type	Bond distance (Å)
ASP 251	Hydrophobic	9.11
LEU 252	Hydrophobic	7.57
ARG 233	Hydrophobic	8.61
LYS 254	Hydrophobic	9.98
PHE 319	Hydrophobic	10.3
VAL 318	Hydrophobic	9.44
ALA 317	Hydrophobic	10.4
LEU 313	Polar	6.98
MET 259	Polar	7.01
ASN 258	Polar	8.12

**FIGURE 7:**
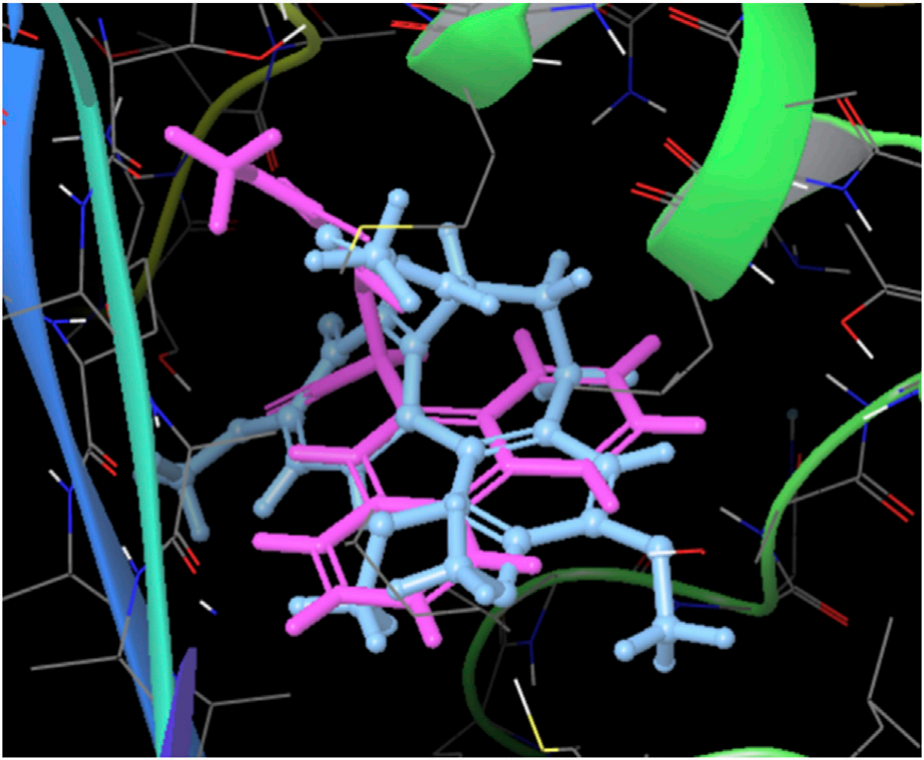
Superposed spatial distributions of colchicine and compound **12E** in the formation of complexes.

Additionally, it is worth highlighting that both molecules, colchicine and compound **12E**, engage in the formation of complexes that exhibit comparable spatial distributions, as illustrated in Figure 7. The spatial congruence in their complex formations suggests a potential similarity in their binding mechanisms and affinities for specific regions on the tubulin structure. This could potentially explain the enhanced stability of the tubulin-compound **12E** complex.

## 4 Discussion

In the realm of antiproliferative agents, this groundbreaking study on cyanomethyl vinyl ether derivatives not only unveils novel compounds but also propels the frontier of cancer research. The synthesis, employing a cutting-edge organocatalytic cyanovinylation methodology, not only underscores the accessibility but also highlights the versatility of these compounds in the pursuit of effective antiproliferative interventions.

Biological evaluations have unearthed compelling cytotoxic activities, particularly noteworthy in the SKOV3 ovarian carcinoma cells, where compounds **5E** and **7E** exhibited pronounced efficacy. The nuanced exploration of the Structure-Activity Relationship (SAR) provides crucial insights, emphasizing the pivotal role of the *E* isomer and specific substitutions in steering the biological activity of these derivatives.

Concluding from the SAR study, the geometry of the double bond of the cyanovinyl ethers is definitely important, being the *E* stereochemistry of the double bond a relevant structural feature giving the most biologically active compounds, even crucial in several cases for activity to be observed. Another important structural point to note in these compounds is also the R^2^ substituent, more interesting those with low number of carbon atoms, in particular the most active ones with methyl group. With respect to the R^1^ substituent (in position α with respect to the cyano group), the influence of groups with fused aromatic rings such as phenanthrene is noteworthy. Therefore, **12E** derivative would be most interesting one from a biological point of view.

Computational assays forecast a promising future for these compounds, positioning them as potential anticancerous agents. The Absorption, Distribution, Metabolism, and Excretion (ADME) studies paint a favorable picture, suggesting not only efficacy but also safety in their pharmacokinetic profile. The meticulous adherence to Lipinski’s Rule of Five further accentuates the drug-like properties of these compounds.

Molecular docking studies delve into the atomic-level interactions, revealing the profound affinity of compound **12E** with tubulin (with the best gScore value). The diverse amino acid interactions, including weak polar interactions with Met259 and Leu313, and robust polar interactions with Thr314 and Val315, underscore the multifaceted stability of the tubulin-compound **12E** complex. This interactional richness potentially translates into enhanced efficacy as an inhibitory agent.

Moreover, the spatial congruence in complex formations with colchicine provides a glimpse into potential mechanistic similarities, enriching our understanding of their binding mechanisms and affinities for specific regions on the tubulin structure.

In summary, this work not only introduces a promising cohort of compounds with substantial cytotoxic potential but also advances our comprehension of their structural-functional dynamics. These findings lay a robust foundation for future drug development endeavors, heralding a new chapter in the pursuit of efficacious antiproliferative therapies.

## Data Availability

The raw data supporting the conclusion of this article will be made available by the authors, without undue reservation.
