# Supplementary material for: Synthesis, biological and computational evaluation of novel cyanomethyl vinyl ether derivatives
Source: Front Pharmacol. 2024 Mar 4;15:1344042. doi: 10.3389/fphar.2024.1344042 (PMC10944868; doi:10.3389/fphar.2024.1344042)
Supplement: Supplementary file 1 [file Table1.DOCX]

Supplementary Material

# RMN Spectra

# Table

| Compound | Log S (log mol/L) | Caco-2 perm. (log Paap in 10-6 cm/s) | Int. Abs. (% abs) | VDss (L/Kg) | Fract. Unb. (Fu.) | BBB permeability (log BB) | CYP1A2 inhibitor | CYP2C19 inhibitor | CYP2C9 inhibitor | CYP2D6 inhibitor | CYP3A4 inhibitor | Total Clearence Numeric (log mL/min/kg) |
| --- | --- | --- | --- | --- | --- | --- | --- | --- | --- | --- | --- | --- |
| **1** | -0.757 | 1.241 | 100 | -0.328 | 0.657 | -0.28 | No | No | No | No | No | 0.949 |
| **2** | -2.706 | 1.441 | 95.66 | -0.143 | 0.439 | -0.179 | No | No | No | No | No | 1.863 |
| **3** | -3.178 | 1.339 | 96.648 | -0.181 | 0.22 | -0.342 | Yes | No | No | No | No | 0.914 |
| **4** | -3.469 | 1.339 | 96.412 | -0.275 | 0.182 | -0.177 | No | No | No | No | No | 0.89 |
| **5** | -2.567 | 1.296 | 97.053 | -0.291 | 0.302 | -0.138 | Yes | No | No | No | No | 0.882 |
| **6** | -3.102 | 1.269 | 98.192 | -0.367 | 0.319 | -0.449 | No | No | No | No | No | 0.853 |
| **7** | -4.159 | 1.365 | 94.971 | -0.334 | 0.263 | -0.185 | Yes | No | No | No | No | 0.427 |
| **8** | -2.264 | 1.312 | 96.557 | -0.367 | 0.343 | -0.042 | Yes | No | No | No | No | 0.662 |
| **9** | -2.526 | 1.318 | 97.113 | -0.171 | 0.322 | -0.022 | Yes | No | No | No | No | 0.852 |
| **10** | -2.598 | 1.301 | 95.722 | -0.357 | 0.362 | -0.186 | Yes | No | No | No | No | 0.455 |
| **11** | -4.944 | 1.356 | 96.911 | 0.193 | 0 | -0.16 | Yes | Yes | Yes | No | No | 0.739 |
| **12** | -4.723 | 1.425 | 96.44 | 0.294 | 0 | -0.096 | Yes | Yes | Yes | No | No | 0.835 |
